# Immune Profiling of Medullary Thyroid Cancer—An Opportunity for Immunotherapy

**DOI:** 10.3390/genes12101534

**Published:** 2021-09-28

**Authors:** Kinga Hińcza-Nowak, Artur Kowalik, Agnieszka Walczyk, Iwona Pałyga, Danuta Gąsior-Perczak, Agnieszka Płusa, Janusz Kopczyński, Magdalena Chrapek, Stanisław Góźdź, Aldona Kowalska

**Affiliations:** 1 Department of Molecular Diagnostics, Holycross Cancer Centre, 25-734 Kielce, Poland; arturko@onkol.kielce.pl; 2 Endocrinology Clinic, Holycross Cancer Centre, 25-734 Kielce, Poland; a.walczyk@post.pl (A.W.); iwonapa@tlen.pl (I.P.); danutagp@o2.pl (D.G.-P.); aldonako@onkol.kielce.pl (A.K.); 3 Division of Medical Biology, Institute of Biology, Jan Kochanowski University, 25-406 Kielce, Poland; 4 Collegium Medicum, Jan Kochanowski University, 25-319 Kielce, Poland; stanislawgo@onkol.kielce.pl; 5 Surgical Pathology, Holycross Cancer Centre, 25-734 Kielce, Poland; agnieszkapl@onkol.kielce.pl (A.P.); januszko@onkol.kielce.pl (J.K.); 6 Faculty of Natural Sciences, Jan Kochanowski University, 25-406 Kielce, Poland; Magdalena.Chrapek@ujk.edu.pl; 7 Clinical Oncology, Holycross Cancer Centre, 25-734 Kielce, Poland

**Keywords:** medullary thyroid cancer, immunotherapy, CD276

## Abstract

Medullary thyroid cancer (MTC) is a rare malignancy that arises from calcitonin-producing C-cells. Curative treatment for patients with metastatic MTC is challenging. Identifying the mechanisms by which cancer cells inhibit the activity of immune cells provides an opportunity to develop new therapies that restore anticancer activity. Little is known about the immunological phenomena underlying MTC. Here, we examined the expression profile of 395 genes associated with MTC. The study included 51 patients diagnosed with MTC at a single center. Bioinformatical analysis revealed that CD276 expression in MTC cells was at least three-fold higher than that in normal tissue. The expression of CD276 showed a weak but statistically significant positive correlation with tumor diameter, but we did not find a significant association between CD276 expression and other histopathological clinical factors, or the response to initial therapy. A search of published data identified the monoclonal antibody (inhibitor) enoblituzumab as a potential drug for patients diagnosed with MTC overexpressing CD276.

## 1. Introduction

Medullary thyroid cancer (MTC), a rare and unique malignancy that arises from calcitonin-producing C-cells, accounts for 3–5% of all thyroid cancers. About 75% of MTC cases occur sporadically; the remaining cases arise in a hereditary pattern as a component of the type 2 multiple endocrine neoplasia syndromes MEN2A and MEN2B, and the related syndrome familial MTC (FMTC). A germline activating mutation in the *RET* protooncogene occurs in nearly all patients with hereditary MTC, whereas a somatic *RET* mutation occurs in approximately 50% of sporadic tumors [1,2,3,4,5]. Molecular studies of MTC have identified *RET*, *HRAS*, and *KRAS* mutations as the major drivers of oncogenesis (*RET*, *HRAS*, and *KRAS* mutations are identified in about 90% of cases). Exome sequencing of sporadic MTCs confirm that *RET* and *RAS* are the dominant drivers. No common recurrent driver mutations other than *RET*, *HRAS*, and *KRAS* have been detected in the MTC exome [6]. Moreover, sporadic MTCs lacking somatic *RET* mutations harbor somatic mutations in *HRAS*, *KRAS*, or *NRAS* [7,8].

The disease course is characterized by high heterogeneity of clinical behavior. MTC diagnosed at the stage of thyroid-restricted disease can be cured by excision of the thyroid. In such cases, the prognosis is good, and the 5-year survival rate is 98% [9]. However, in some cases, patients may show evidence of cervical lymph node metastases or distant metastases at the time of diagnosis. Curative treatment for patients with metastatic MTC remains problematic [10]. Locally advanced forms are treated with teleradiotherapy, whereas metastatic disease is treated with tyrosine kinase inhibitors cabozantinib and vandetanib [11,12,13]. Both of these drugs increase progression-free survival. A recent study suggests that the highly selective inhibitor receptor tyrosine kinase RET is a new treatment option for patients harboring mutations in the *RET* protooncogene. Unlike other drugs that block several proteins at once, selpercatinib targets only the mutated RET protein, thereby reducing the risk of side effects [14,15]. However, many patients become refractory to these drugs. Therefore, there is a need to identify new treatments for metastatic MTC. Human tumors should be considered as complex tissues that include various cellular and noncellular components associated with cancer cells. These components create the so-called tumor microenvironment (TME). The TME includes the extracellular matrix, mesenchymal cells, immunoinflammatory cells, blood, and lymphatic vessels [16]. Communication between cancer cells and the TME has a marked effect on oncogenesis and tumor growth, progression, and metastasis. Tumor cells actively remodel the preexisting stroma to create a new microenvironment with pro-inflammatory characteristics that are conducive to survival [17]. The composition of the stroma associated with thyroid cancer changes according to the histotype. MTC cells display a desmoplastic stromal reaction that correlates with aggressiveness and lymph node metastasis [18]. Recent data suggest that only MTC with a desmoplastic stromal reaction develops regional lymph node metastasis16. An important component of the TME is immunoinflammatory cells. Cancer development and progression are affected profoundly by the immune system. Factors produced by cancer cells and other microenvironmental factors in the TME favor the flux of immune cells around tumors [19]. Infiltration by immune cells, and their interactions within the TME, affect tumor development and progression; as such, they are the subject of intense investigation. Identifying the mechanisms by which cancer cells inhibit the activity of immune cells provides an opportunity to develop new therapies that restore anticancer activity [20,21]. Immune surveillance is a process by which cancer cells are recognized and eliminated by the immune system; however, tumors often escape immune-mediated elimination via different mechanisms [22]. Little is known about the immunological phenomena accompanying MTC. Therefore, it is important to identify molecular changes that are conducive to formation of neoantigens that initiate immune responses; such neoantigens may be potent therapeutic targets. Diagnostic panels based on gene expression profiles associated with activation of individual immune cell types are sensitive tools for evaluating the immunological processes accompanying MTC. Tumor cells often demonstrate altered expression of cytokines and chemokines, which promote activity of suppressive immune cell populations; they also express immune checkpoint molecules that inhibit antitumor immune responses [23,24,25]. In this study, we used a panel to assess the expression profile of 395 genes in MTC tissue. This analysis identified a potential candidate for immunotherapy of MTC.

## 2. Results

### 2.1. Characteristics at Presentation and Primary Treatment

Patient demographics and the clinicopathological features of all 51 cases are presented in Table 1. The study group comprised 36 women (70.6%, 36/51) and 15 men (29.4%, 14/51). The median age at diagnosis was 53 years (range, 24–84). The median tumor size was 12 mm (range, 0.5–100). Most patients (70.6%, 36/51) did not have tumor multifocality. Gross type extrathyroidal extension was detected in 7.8% patients (4/51). Angioinvasion was present in 11.8% (6/51) of patients. Lymph node metastases and distant metastases were present in 39.2% (20/51) and 3.9% (2/51) of patients, respectively.

### 2.2. Response to Therapy

The initial response to therapy was evaluated 3 months after surgery. The type of response was classified as excellent, biochemical incomplete, or structural incomplete. An excellent response to primary therapy was observed in 27 patients (52.9%). A biochemical incomplete response to initial therapy was observed in 18 patients (35.3%), while a structural incomplete response was observed in two patients (3.9%). There were four deaths among cases with MTC, but only two cases were MTC-related (Table 2).

### 2.3. Expression of 395 Genes in Tumor and Control Tissues

Affymetrix™ Transcriptome Analysis Console (TAC) (version 4.1) software was used to compare the gene expression profiles between tumor tissue from patients with MTC and those from benign tissue (control). Bioinformatical analysis revealed that CD276 expression by cancer cells was at least three-fold higher than that in normal tissue (fold change; 3.16). In addition, the analysis had a low false discovery rate (FDR *p*-value; 0.000015) (Table S1). CD276 was selected for further investigation based on its significant role in the course of other types of cancer and on a lack of literature reports about its role in development and course of MTC. Thus, the expression of CD276 was subjected to further analysis (Table S2).

### 2.4. Relationship between CD276 Expression and Histopathologic Factors

**Figure 1:**
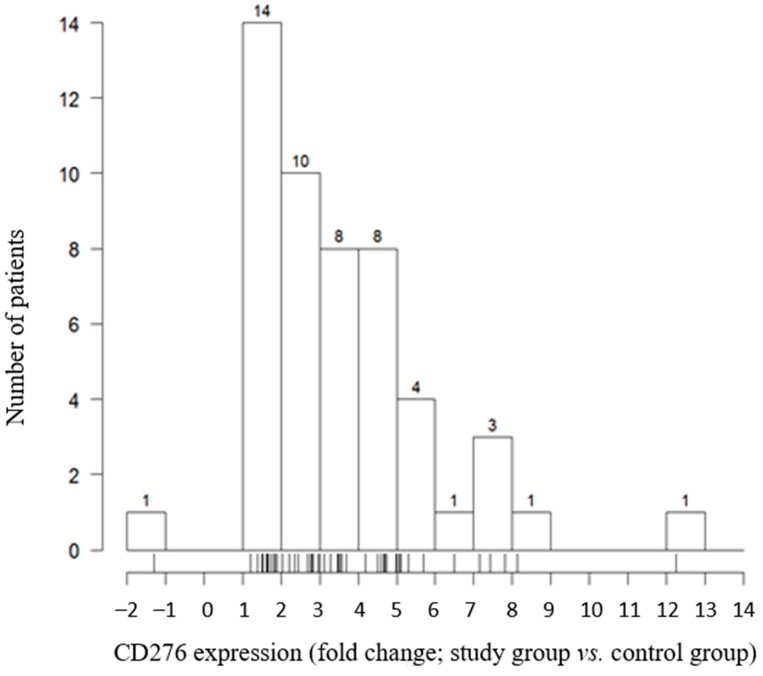
CD276 expression by tumor cells.

In >85% of patients, the expression of CD276 was between 1 and 6 times higher than that in controls (44/51 (86.2%)), whereas in almost half of patients it was between 1 and 3 times higher (24/51 (47.1%)). Only one patient showed higher expression in normal tissue than in the tumor. Overall, the CD276 expression in tumor cells was significantly higher than that in healthy tissue (*p* < 0.0001; Figure 1).

**Figure 2:**
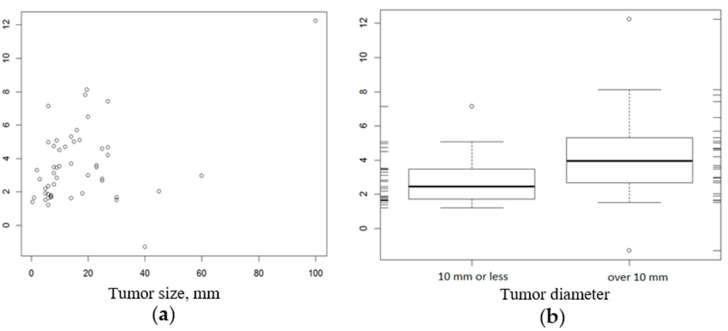
Relationship between CD276 expression by tumor cells and tumor size, mm (**a**); range of tumor diameter (**b**).

We also assessed the relationship between CD276 expression in tumor cells and clinical factors such as the response to initial therapy and overall mortality. The expression of CD276 showed a weak but statistically significant positive correlation with tumor diameter (Spearman’s rank correlation coefficient = 0.31, *p* = 0.028; Figure 2). However, we did not find a significant association between CD276 expression and other histopathological clinical factors, or the response to initial therapy (Table 3 and Table 4).

**Figure 3:**
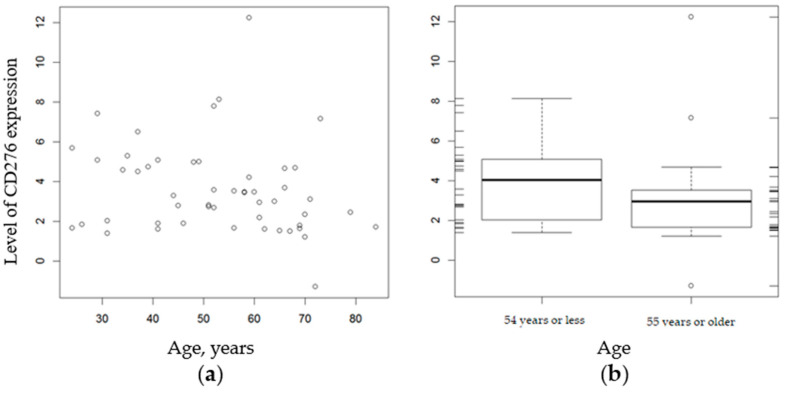
Relationship between CD276 expression by tumor cells and age, years (**a**); range of age (**b**).

In addition, a statistical analysis comparing the relationship between CD276 expression and gender (Table 5) and age (Table 6) indicated no significant difference. The expression of CD276 showed a weak, statistically insignificant negative correlation with age at diagnosis (Spearman’s rank correlation coefficient = −0.27, *p* = 0.054) (Figure 3).

### 2.5. Potential Therapeutic Targets for MTC

Finally, we scanned the Drug Gene Interaction database DGIdb (http://www.dgidb.org/, accessed on 30 June 2021) to identify a potential drug for patients diagnosed with MTC overexpressing CD276. The DGIdb combines information from the DrugBank (https://www.drugbank.ca/, accessed on 30 June 2021), the Therapeutic Target Database (http://bidd.nus.edu.sg/group/cjttd/, accessed on 30 June 2021), PharmGKB (https://www.pharmgkb.org/, accessed on 30 June 2021), and ClinicalTrials.gov databases (accessed on 30 June 2021). The analysis identified one candidate: the monoclonal antibody (inhibitor) enoblituzumab.

## 3. Discussion

CD276, first identified in 2001 as a member of the B7 ligand family [26], is expressed by antigen-presenting cells, macrophages, and tumor cells, and has inhibitory effects on T-cells; these effects allow tumor cells to evade immune responses [25,27]. Accumulating evidence shows that CD276 is involved in biological processes underlying cancer development, including proliferation, migration, invasion, drug resistance, and metabolism [25,28]. CD276 is overexpressed by several human cancers and its expression correlates with poor outcomes [26]. CD276 is present at low levels in normal tissues but is overexpressed by bladder, breast, cervical, colorectal, esophageal, glioma, kidney, liver, lung, ovarian, pancreatic, prostate, liver, oral squamous cell carcinoma, endometrial cancer, squamous cell carcinoma, and gastric cancer cells [29,30,31,32,33,34,35,36,37,38,39].

B7-H3 is a co-stimulatory molecule involved in immune reactions such as T-cell activation and IFN-γ production [26]. CD276 remains an orphan ligand, but a potential receptor, called TLT-2, has been detected on activated immune cells [40]. A recent study shows that CD276 is overexpressed by both cancer cells and the tumor vasculature [29]. A recent investigation showed that CD276/TLT-2 increases the production of chemokines and pro-inflammatory cytokines by activating phosphorylation of mitogen-activated protein kinase p38 and NF-kappa B p65 [41]. However, these effects were not observed in another study of both human and murine CD276 [42]. Thus, there is insufficient evidence for TLT-2 as a receptor of CD276. This ambiguity may account for the contradictory co-stimulatory and co-inhibitory roles played by the CD276 molecule during immune responses.

The role of CD276 during development and the clinical course of MTC are unknown. In this study, statistical analysis revealed that the expression of CD276 in medullary thyroid tumor cells was significantly higher than that in healthy tissue (*p* < 0.0001). Moreover, assessment of the relationship between CD276 expression in tumor cells and clinical factors demonstrated a weak but statistically significant positive correlation with tumor diameter (Spearman’s rank correlation coefficient = 0.31, *p* = 0.028). As far as we are aware, this is the first report of increased CD276 expression in MTC cells relative to that in normal tissue. The overexpression of this immune checkpoint receptor represents a promising therapeutic target. However, successful targeting would require knowledge about the amount of receptor on the cells and its distribution within tissues [29].

An attractive option is the monoclonal antibody (inhibitor) enoblituzumab, which is a Fc-optimized humanized IgG1 monoclonal antibody that binds to B7-H3 expressed by solid tumors. The CD276 pathway plays a dual role during innate immune responses. One study reports that neuroblastoma cells express CD276 on the surface, which protects them from NK cell-mediated lysis. However, another study argues that CD276 co-stimulates innate immunity by augmenting the release of pro-inflammatory cytokines from LPS-stimulated monocytes/macrophages in both a Toll-like receptor 4- and 2-dependent manner. Published data suggest that CD276 plays an important role in T-cell-mediated adaptive immunity [43,44]. Enoblituzumab is a humanized monoclonal antibody that targets cancer stem cells (CSCs), thereby exerting potential immunomodulating activity. The binding of enoblituzumab to an as-yet-unidentified target expressed on CSCs and differentiated tumor cells may induce an antibody-dependent cell-mediated cytotoxicity response. Weekly doses of enoblituzumab given to mice bearing both renal and bladder carcinoma xenografts resulted in sustained inhibition of tumor growth. So far, enoblituzumab has been evaluated in combination with retifanlimab in a Phase 2 clinical study of a chemotherapy-free regimen for front-line patients with squamous cell carcinoma of the head and neck (SCCHN) and who are PD-L1-positive; it has also been used with tebotelimab in SCCHN patients who are PD-L1 negative [43,45,46]. Shenderov et al. reported using enoblituzumab for the treatment of prostate cancer; enoblituzumab was well tolerated and did not seem to produce as many side effects as other immunotherapy drugs. Moreover prostatectomy samples from men who received enoblituzumab showed an altered TME that suggested enhanced immune infiltration [47].

## 4. Materials and Methods

### 4.1. Patients and Controls

All study procedures were approved by the Institutional Review Board of Jan Kochanowski University, Kielce, Poland (approval number: 12/2020). The study included 51 patients with MTC (47 living and 4 dead patients). A group of 10 benign thyroid tumors was included as a control cohort. All cases and controls were recruited at the Holycross Cancer Centre (HCC) in Kielce. MTC patients were selected from the thyroid cancer database at the Endocrinology Clinic of HCC. Histopathological slides and paraffin blocks of MTC tissues were identified from the archives at the Department of Cancer Pathology. Two pathologists (J.K. and A.P.) verified the MTC diagnosis independently. The medical records of patients were reviewed to obtain demographic information, the histoclinical characteristics of the cancer, treatments, responses to treatment, and clinical course. The comparison group included tissue from patients with benign thyroid tumors and normal thyroid tissue surrounding areas of MTC. Serial sections were cut from paraffin blocks selected by the pathologist; one section per sample was stained with hematoxylin and eosin. An area containing cancer cells was marked on the stained slides. Similarly, the pathologist selected areas on slides of macrodissected benign tumors and normal thyroid tissue. RNA was isolated from these tissues as described below.

### 4.2. Isolation of RNA from Formalin-Fixed, Paraffin-Embedded (FFPE) Samples

The commercially available Maxwell^®^ RSC RNA FFPE Kit, which is dedicated for use on the Maxwell RSC device, was used for RNA isolation. RNA was isolated using the protocol provided by the manufacturer (Promega, Madison, WI, USA). Quality was verified using a Qubit 4 Fluorometer (Thermo Fisher Scientific, Waltham, MA, USA) and a Qubit RNA Assay Kit.

### 4.3. Reverse Transcription

RNA was reverse transcribed to cDNA using the SuperScript™ VILO cDNA Synthesis Kit (Thermo Fisher Scientific, Waltham, MA, USA).

### 4.4. Oncomine Immune Response Research Assay

Isolated RNA was analyzed using the Oncomine Immune Response Research Assay (Thermo Fisher Scientific, Waltham, MA, USA). The Oncomine Immune Response Research Assay allows analysis of expression of 395 genes related to lymphocyte regulation, cytokine signaling, lymphocyte markers, and checkpoint pathways (Table S3). The platform identifies genes associated with adhesion, migration, antigen presentation, antigen processing, apoptosis, B-cell markers, B-cell receptor signaling, checkpoint pathways, chemokine signaling, cytokine signaling, dendritic cells, macrophages, drug targets, helper T-cells, housekeeping, innate immune responses, interferon signaling, leukocyte inhibition, leukocyte migration, lymphocyte activation, lymphocyte development, lymphocyte infiltration, myeloid markers, neutrophils, NK activation, NK-cell markers, PD-1 signaling, proliferation, T-cell differentiation, T-cell receptor signaling, T-cell regulation, TCR co-expression, tumor antigens, tumor markers, type I interferon signaling, and type II interferon signaling. The test allows evaluation of expression of genes involved inneoplastic-immunological interactions, as well as the level of the expression of genes involved in inflammatory signaling pathways.

### 4.5. Emulsion PCR and Sequencing

A library was prepared using the Ion AmpliSeq™ Library Kit Plus and Ion Xpress Barcode Adapters Kit (Thermo Fisher Scientific) and purified using Agencourt AMPure XP (Beckman Coulter Genomics, Brea, CA, USA). The concentration of the libraries was measured using real-time quantitative PCR (qRT-PCR), the Ion Library TaqMan™ Quantitation Kit (Thermo Fisher Scientific), and the Rotor-Gene Q apparatus (Qiagen, Hilden, Germany). Based on the values obtained from qRT-PCR, libraries prepared using the Oncomine Immune Response Research Assay Kit (Thermo Fisher Scientific) were diluted to 100 pM. Next, IonChef (Thermo Fisher Scientific) sets Ion 520, Ion 530 Kit-Chef, and the Ion 530™ Chip Kit (Thermo Fisher Scientific) were used for emulsion PCR to enrich the samples and to load the appropriate chips. Sequencing was performed using the Ion GeneStudio S5 Prime Sequencer (Thermo Fisher Scientific), according to the manufacturer’s instructions.

### 4.6. Bioinformatics Analysis

Data obtained from RNA sequencing using the Oncomine Immune Response Research Assay (Thermo Fisher Scientific) were analyzed using the program that is integral to the Ion Torrent Server (ImmuneResponseRNA) and the Affymetrix™ Transcriptome Analysis Console (TAC) 4.1 software (Thermo Fisher Scientific). The ImmuneResponseRNA program allows for basic qualitative evaluation of the test results based on the number of readings obtained and the corresponding graphs. Affymetrix™ Transcriptome Analysis Console (TAC) 4.1 software was used to compare the gene expression profiles in tumor tissue from patients with MTC with those in benign tissue from the control group.

### 4.7. Statistical Analysis

The difference (DIFF) in expression of CD276 between tumor and normal tissue was calculated for each patient. Continuous data were expressed as the mean, standard deviation, median, quartile, and range (minimum and maximum). Categorical data were expressed as numbers and percentages. The normality of the distributions was checked using the Shapiro–Wilk test. Due to non-normality, the Mann–Whitney test and the Wilcoxon signed rank test were used to compare unrelated and related groups, respectively. Spearman’s rank correlation coefficients were calculated to assess the bivariate relationship between DIFF and tumor diameter or calcitonin levels. A two-tailed *p*-value < 0.05 was considered statistically significant. All statistical analyses were performed using the R software package version 4.0.3.

## 5. Conclusions

The immune response to cancer is a complicated process that involves many factors and pathways, which organically affect each other. Antigens expressed by tumor cells, the number of immune cells, antigen recognition pathways, and the interaction between tumor cells and immune cells all play roles. The data presented herein provide insight into the MTC microenvironment and identify an alternative immune checkpoint, which may facilitate the development of new treatments.

## Figures and Tables

**Table 1:** Characteristics of the study group.

Feature	Total (*n* = 51)
Gender, *n* (%)	
Female	36 (70.6%)
Male	15 (29.4%)
Median age at diagnosis, years (Q1–Q3; range)	53.0 (41.0, 65.5; 24–84)
Median tumor size, mm (Q1–Q3; range)	12.0 (6.5, 23.0; 0.5–100)
Multifocality, *n* (%)	
No	36 (70.6%)
Yes	17 (33.3%)
Extrathyroidal extension, *n* (%)	
No	47 (92.2%)
Yes	4 (7.8%)
Angioinvasion, *n* (%)	
No	45 (88.2%)
Yes	6 (11.8%)
Tumor stage, *n* (%)	
T1a	16 (31.4%)
T1am	8 (15.7%)
T1b	9 (17.6%)
T1bm	3 (5.9%)
T2	6 (11.8%)
T2m	4 (7.8%)
T3	3 (5.9%)
T3m	2 (3.9%)
Node stage, *n* (%)	
N0	31 (60,7,%)
N1a	6 (11.8%)
N1b	14 (27.5%)
Distant metastasis, *n* (%)	
M0	49 (96,1%)
M1	2 (3.9%)

T—tumor; N—node; M—metastasis.

**Table 2:** Initial response to therapy.

**Initial Response to Therapy**	***n* (%)**
Excellent	27 (52.9%)
Biochemical incomplete response	18 (35.3%)
Structural incomplete response	2 (3.9%)
**Death**	***n* (%)**
No	47 (92.2%)
MTC-unrelated	2 (3.9%)
MTC-related	2 (3.9%)

MTC: medullary thyroid cancer.

**Table 3:** Relationship between CD276 expression and histopathological and clinical factors.

CD276 Expression	Feature		*p*-Value
	**Tumor Size, mm**		
	10 mm or less (*n* = 25)	Over 10 mm (*n* = 26)	
			0.0205
Mean (SD)	2.89 (1.47)	4.27 (2.71)	
Median (Q1, Q3)	2.44 (1.72, 3.47)	3.94 (2.71, 5.25)	
Range	1.21–7.15	−1.29–12.24	
	**Multifocality**		
	No (*n* = 36)	Yes (*n* = 15)	
			0.2265
Mean (SD)	3.88 (2.32)	2.91 (2.08)	
Median (Q1, Q3)	3.46 (2.23, 4.99)	2.20 (1.69, 4.62)	
Range	1.21–12.24	−1.29–7.42	
	**Angioinvasion**		
	No (*n* = 45)	Yes (*n* = 6)	
			0.5201
Mean (SD)	3.48 (1.85)	4.42 (4.55)	
Median (Q1, Q3)	3.00 (1.90, 4.66)	4.29 (2.12, 5.25)	
Range	1.21–8.13	−1.29–12.24	
	**Central Lymph Nodes**		
	No (*n* = 45)	Yes (*n* = 6)	
			0.3571
Mean (SD)	3.71 (2.19)	2.71 (2.95)	
Median (Q1, Q3)	3.29 (1.90, 4.74)	1.96 (1.73, 3.88)	
Range	1.21–12.24	−1.29–7.42	
	**Lateral Lymph Nodes**		
	No (*n* = 37)	Yes (*n* = 14)	
			0.1542
Mean (SD)	3.25 (1.93)	4.49 (2.89)	
Median (Q1, Q3)	2.83 (1.72, 4.69)	3.50 (2.75, 5.14)	
Range	−1.29–7.80	1.61–12.24	
	**Node Stage**		
	N0 (*n* = 31)	N1 (*n* = 20)	
			0.4873
Mean (SD)	3.35 (1.72)	3.96 (2.95)	
Median (Q1, Q3)	3.00 (1.75, 4.71)	3.48 (1.90, 4.82)	
Range	1.21–7.80	−1.29–12.24	

**Table 4:** Relationship between CD276 expression and response to initial therapy.

Level CD276 Expression	Response to Initial Therapy, *n* (%)					*p*-Value
	Remission (*n* = 27)	Biochemical Persistent Disease (*n* = 18)	Structural Persistent Disease (*n* = 2)	Death MTC-Related (*n* = 2)	Death MTC-Unrelated (*n* = 2)	
Mean (SD)	3.27 (1.73)	2.89 (1.57)	5.24 (3.62)	10.19 (2.91)	5.95 (1.70)	0.457
Median (Q1, Q3)	3.00 (1.65, 4.60)	2.90 (1.90, 3.52)				
Range	1.21–7.42	−1.29–5.30	2.68–7.80	8.13–12.24	4.74–7.15	

**Table 5:** Relationship between CD276 expression and gender.

Level CD276 Expression	Gender			*p*-Value
	10 mm or Less (*n* = 25)	Over 10 mm (*n* = 26)	Total (*n* = 51)	
Mean (SD)	3.47 (2.40)	3.89 (2.00)	3.59 (2.28)	0.4821
Median (Q1, Q3)	2.88 (1.82, 4.80)	3.47 (2.81, 4.62)	3.12 (1.88, 4.71)	
Range	−1.29–12.24	1.51–8.13	−1.29–12.24	

**Table 6:** Relationship between CD276 expression and age.

Level CD276 Expression	Age, Years		*p*-Value
	54 Years or Less (*n* = 26)	55 Years or Older (*n* = 25)	
Mean (SD)	4.04 (2.02)	3.12 (2.47)	0.0511
Median (Q1, Q3)	4.04 (2.19, 5.09)	2.96 (1.67, 3.53)	
Range	1.39–8.13	−1.29–12.24	

## Data Availability

Data are available on request due to restrictions privacy and ethical.

## Supplementary Materials

The following are available online at https://www.mdpi.com/article/10.3390/genes12101534/s1, Table S1: Genes with more than doubled expression levels between the study and control groups, Table S2: Results from the Oncomine for CD276 gene and the patients clinical characteristics, Table S3: Gene list from Oncomine Immune Response Research Assay.
