# Supplementary material for: Immune Profiling of Medullary Thyroid Cancer—An Opportunity for Immunotherapy
Source: Genes (Basel). 2021 Sep 28;12(10):1534. doi: 10.3390/genes12101534 (PMC8536131; doi:10.3390/genes12101534)
Supplement: Supplementary file 1 [file genes-12-01534-s001.zip › Table S1_Genes with more than doubled expression levels between the study and control groups..pdf]

**Gene Symbol**	**Fold Change (linear) (Study group vs, control)**	**FDR p-value (Study group vs, control)**	**Description**	**Gene Function**
MPO	2,08	0,292303	myeloperoxidase	Myeloid marker
NOTCH3	2	0,00022	notch_3	Tumor marker
BCL2	2,94	0,000381	B-cell_CLL_lymphoma_2	Apoptosis
POLR2A	2,01	0,00041	polymerase_RNA_II_subunit_A	Housekeeping
CD276	3,16	0,000015	CD276_molecule	Checkpoint pathway
GAGE2C,GAGE2A,GAGE2E	2,05	0,219258	G_antigen_2C	Tumor antigen
HLA-A	7,18	0,569056	major_histocompatibility_complex_class_I_A	Antigen processing
HLA-C	3,06	0,650337	major_histocompatibility_complex_class_I_C	Antigen processing
TNFRSF14	2,28	0,000992	tumor_necrosis_factor_receptor_superfamily_member_14	Checkpoint pathway
MTOR	2,04	0,000031	mechanistic_target_of_rapamycin	PD-1 signaling, tumor marker
TNFSF18	2,24	0,070011	tumor_necrosis_factor_superfamily_member_18	Checkpoint pathway
HLA-B	7,78	0,020063	major_histocompatibility_complex_class_I_B	Antigen processing
ICOSLG	2,12	0,000264	inducible_T-cell_costimulator_ligand	Checkpoint pathway
IKZF4	3,63	0,000357	IKAROS_family_zinc_finger_4	Lymphocyte development
NCAM1	2,71	0,000197	neural_cell_adhesion_molecule_1	Adhesion,migration
