# Supplementary material for: Immune Profiling of Medullary Thyroid Cancer—An Opportunity for Immunotherapy
Source: Genes (Basel). 2021 Sep 28;12(10):1534. doi: 10.3390/genes12101534 (PMC8536131; doi:10.3390/genes12101534)
Supplement: Supplementary file 1 [file genes-12-01534-s001.zip › Table S2_Results from the Oncomine for CD276 gene and the patients clinical characteristics.pdf]

Case number	CD276 Fold Change (linear) (Study group vs, control)	Tumor Size, mm	Extrathyroidal extension No-0 Yes-1	Angioinvasion No-0 Yes-1	Lymph Nodes No-0 Yes-1	Tumor stage	Node stage	Distant metastasis	Type of MTC sporadic-0 familial-1	Initial Response to Therapy Excellent-0 Biochemical incomplete response-1 Structural incomplete response-2 Death MTC-related-3 Death MTC-unrelated-4	Follow up (years)
1	3,29	2	0	0	0	T1a	N0	M0	0	0	4
2	3	20	0	0	0	T1b	N0	M0	0	0	5
3	4,2	27	0	0	0	T2	N0	M0	0	0	6
4	4,5	10	0	0	1	T1a	N1	M0	0	0	6
5	3,57	23	0	0	0	T2	N0	M0	0	0	7
6	1,9	18	1	0	1	T3	N1a	M0	0	1	11
7	2,79	25	0	0	0	T2	N0	M0	0	0	2
8	7,15	6	0	0	0	T1a	N0	M0	0	4	2
9	4,66	27 and 12	0	0	1	T2m	N1b	M0	1	1	6
10	4,74	8 and 4	0	0	0	T1m	N0	M0	1	4	12
11	4,59	25 and 9	0	0	1	T2m	N1b	M0	1	1	4
12	1,39	0,5	0	0	0	T1a	N0	M0	0	0	3
13	7,8	19	0	0	0	T1b	N0	M0	0	2	17
14	8,13	19,55	0	0	1	T3	N1b	M0	0	3	1
15	7,42	27 and 13	0	0	1	T2m	N1	M0	1	0	11
16	4,98	6	0	0	0	T1a	N0	M0	0	0	5
17	5,69	16,1	0	0	0	T1bm	N0	M0	0	0	11
18	5	15	0	0	0	T1b	N0	M0	0	0	6
19	12,24	100	0	1	1	T3	N1	M1	0	3	1
20	4,69	12	0	0	0	T1b	N0	M0	0	0	9
21	5,3	14	1	1	1	T1bm	N1b	Mx	1	1	1
22	6,5	20	0	0	1	T1b	N1b	M0	0	0	1
23	3,53	10	0	0	1	T1b	N1b	M0	1	1	12
24	3,12	8	0	0	0	T1a	N0	M0	0	0	1

25	3,47	8	0	0	1	T1a	N1b	M0	0	1	9
26	5,09	17	0	1	0	T1b	N0	M0	0	0	2
27	2,03	45 and 30	0	0	1	T3m	N1a	M0	1	1	6
28	1,21	6	0	0	0	T1a	N0	M0	0	0	10
29	2,96	x	0	0	1	T3	N1b	Mx	1	1	12
30	3,48	23	0	1	1	T2	N1b	M0	0	1	2
31	3,45	9	0	0	0	T1a	N0	M0	0	1	14
32	2,75	3	0	0	0	T1am	N0	M0	0	1	9
33	2,44	8	0	0	x	T1a	Nx	M0	0	0	8
34	2,34	6	0	0	0	T1a	N0	M0	0	0	7
35	1,53	5	0	0	x	T1a	Nx	M0	0	0	7
36	3,68	14	0	0	0	T1b	N0	M0	0	0	6
37	1,79	7	0	0	x	T1a	NX	M0	0	1	5
38	1,64	1+hyperplasia	0	0	x	T1a	Nx	M0	0	0	5
39	5,08	9 and 7	0	0	0	T1am	N0	M0	1	1	12
40	2,2	4 and 5 and 1,5	0	0	0	T1am	N0	M0	1	0	4
41	2,83	9 and 2 and 1	0	0	0	T1am	N0	M0	1	1	3
42	2,68	25	0	0	1	T12	N1b	M1	1	2	2
43	1,72	7 and 1	0	0	0	T1am	N0	M0	0	0	2
44	1,67	30 and 2	1	1	0	T2m	N0	M0	0	0	2
45	1,51	30	0	0	0	T2	N0	M0	0	0	2
46	1,61	14 and 4	0	0	0	T1bm	N0	M0	1	0	2
47	1,61	6	0	0	0	T1a	N1b	M0	0	0	2
48	1,9	4 and 5	0	0	1	T1am	N1b	M0	1	1	1
49	-1,29	40 and 19 + hyperplasia	1	1	1	T3m	N1a	M0	1	1	1
50	1,67	7 and 4	0	0	1	T1am	N1a	M0	1	1	1
51	1,85	6	0	0	1	T1a	N1b	M0	0	1	3
