# Supplementary material for: Immune Profiling of Medullary Thyroid Cancer—An Opportunity for Immunotherapy
Source: Genes (Basel). 2021 Sep 28;12(10):1534. doi: 10.3390/genes12101534 (PMC8536131; doi:10.3390/genes12101534)
Supplement: Supplementary file 1 [file genes-12-01534-s001.zip › Table S3_Gene list_Oncomine Immune Response Research Assay.pdf]

Gene	GENE_FUNCTION
ABCF1	Housekeeping
ADGRE5	Adhesion,migration
ADORA2A	Checkpoint_pathway
AIF1	Macrophage
AKT1	Tumor_marker
ALOX15B	Macrophage
ARG1	Myeloid_marker
AXL	Innate_immune_response
B3GAT1	NK_activation
BAGE	Tumor_antigen
BATF	Helper_T_cells
BCL2	Apoptosis
BCL2L11	Apoptosis
BCL6	Type_II_interferon_signaling
BRCA1	Tumor_marker
BRCA2	Tumor_marker
BST2	Type_I_interferon_signaling
BTLA	Checkpoint_pathway
BUB1	Proliferation
C10orf54	Checkpoint_pathway
C1QA	Innate_immune_response
C1QB	Innate_immune_response
CA4	Neutrophil
CBLB	T_cell_receptor_signaling
CCL17	Chemokine_signaling
CCL18	Lymphocyte_infiltrate
CCL2	Lymphocyte_infiltrate
CCL20	Chemokine_signaling
CCL21	Lymphocyte_infiltrate
CCL22	Chemokine_signaling
CCL3	Lymphocyte_infiltrate
CCL4	Lymphocyte_infiltrate
CCL5	Lymphocyte_infiltrate
CCNB2	Proliferation
CCR1	Cytokine_signaling
CCR2	Helper_T_cells
CCR4	Chemokine_signaling
CCR5	Lymphocyte_infiltrate
CCR6	Chemokine_signaling
CCR7	TCR_coexpression
CD14	Dendridic_cell,macrophage
CD160	Checkpoint_pathway
CD163	Macrophage
CD19	B_cell_marker
CD1C	Antigen_presentation
CD1D	Antigen_presentation
CD2	Lymphocyte_infiltrate
CD209	Dendridic_cell,macrophage
CD22	B_cell_marker

CD226	Adhesion,migration
CD244	Checkpoint_pathway
CD247	TCR_coexpression
CD27	Drug_target
CD274	Checkpoint_pathway
CD276	Checkpoint_pathway
CD28	Checkpoint_pathway
CD33	Myeloid_marker
CD37	Lymphocyte_infiltrate
CD38	Adhesion,migration
CD3D	TCR_coexpression
CD3E	TCR_coexpression
CD3G	TCR_coexpression
CD4	Helper_T_cells
CD40	Drug_target
CD40LG	T_cell_receptor_signaling
CD44	Adhesion,migration
CD47	Adhesion,migration
CD48	Checkpoint_pathway
CD52	Lymphocyte_infiltrate
CD53	Adhesion,migration
CD6	TCR_coexpression
CD63	Lymphocyte_infiltrate
CD68	Macrophage
CD69	Checkpoint_pathway
CD70	Drug_target
CD74	Antigen_processing
CD79A	B_cell_receptor_signaling
CD79B	B_cell_receptor_signaling
CD80	Checkpoint_pathway
CD83	Antigen_presentation
CD86	Checkpoint_pathway
CD8A	TCR_coexpression
CD8B	TCR_coexpression
CDK1	Proliferation
CDKN2A	Tumor_marker
CDKN3	Proliferation
CEACAM1	Checkpoint_pathway
CEACAM8	Myeloid_marker
CIITA	Type_II_interferon_signaling
CLEC4C	Dendridic_cell
CMKLR1	Dendridic_cell,macrophage
CORO1A	Lymphocyte_infiltrate
CRTAM	TCR_coexpression
CSF1R	Cytokine_signaling
CSF2RB	Cytokine_signaling
CTAG1B	Tumor_antigen
CTAG2	Tumor_antigen
CTLA4	Drug_target
CTSS	Lymphocyte_infiltrate

CX3CL1	Type_II_interferon_signaling
CX3CR1	Lymphocyte_infiltrate
CX3CR1	Lymphocyte_infiltrate
CX3CR1	Lymphocyte_infiltrate
CX3CR1	Lymphocyte_infiltrate
CXCL1	Chemokine_signaling
CXCL10	Type_II_interferon_signaling
CXCL11	Type_II_interferon_signaling
CXCL13	Type_II_interferon_signaling
CXCL8	Cytokine_signaling
CXCL9	Type_II_interferon_signaling
CXCR2	Chemokine_signaling
CXCR3	Chemokine_signaling
CXCR4	Lymphocyte_infiltrate
CXCR5	Type_II_interferon_signaling
CXCR6	Lymphocyte_infiltrate
CYBB	Type_II_interferon_signaling
DDX58	Interferon_signaling
DGAT2	Neutrophil
DMBT1	Innate_immune_response
EBI3	T_cell_regulation
EFNA4	Tumor_marker
EGFR	Tumor_marker
EGR2	T_cell_differentiation
EGR3	Tumor_marker
EIF2AK2	Type_II_interferon_signaling
ENTPD1	Checkpoint_pathway
EOMES	Checkpoint_pathway
FAS	B_cell_receptor_signaling
FASLG	Type_II_interferon_signaling
FCER1G	Lymphocyte_infiltrate
FCGR1A	B_cell_marker
FCGR2B	B_cell_marker
FCGR3A	Macrophage
FCGR3B	NK_activation
FCRLA	B_cell_marker
FOXM1	Proliferation
FOXO1	PD-1_signaling,tumor_marker
FOXP3	T_cell_regulation
FUT4	Myeloid_marker,stem_cell
FYB	Lymphocyte_infiltrate
G6PD	Housekeeping
GADD45GIP1	Apoptosis
GAGE1,GAGE12I,GAGE12F	Tumor_antigen
GAGE10	Tumor_antigen
GAGE12J	Tumor_antigen
GAGE13	Tumor_antigen
GAGE2C,GAGE2A,GAGE2E	Tumor_antigen
GATA3	Helper_T_cells
GBP1	Type_II_interferon_signaling

GNLY	NK_activation
GPR18	TCR_coexpression
GRAP2	TCR_coexpression
GUSB	Housekeeping
GZMA	Lymphocyte_infiltrate
GZMB	Lymphocyte_infiltrate
GZMH	Lymphocyte_infiltrate
GZMK	Lymphocyte_infiltrate
HAVCR2	Checkpoint_pathway
HERC6	Dendridic_cell
HGF	Cytokine_signaling
HIF1A	PD-1_signaling,tumor_marker
HLA-A	Antigen_processing
HLA-B	Antigen_processing
HLA-C	Antigen_processing
HLA-DMA	Antigen_processing
HLA-DMB	Antigen_processing
HLA-DOA	Antigen_processing
HLA-DOB	Antigen_processing
HLA-DPA1	Antigen_processing
HLA-DPB1	Antigen_processing
HLA-DQA1	Antigen_processing
HLA-DQA2	Antigen_processing
HLA-DQB2	Antigen_processing
HLA-DRA	Antigen_processing
HLA-DRB1	Antigen_processing
HLA-E	Antigen_processing
HLA-F	Antigen_processing
HLA-F-AS1	Antigen_processing
HLA-G	Antigen_processing
HMBS	Housekeeping
ICAM1	Type_II_interferon_signaling
ICOS	Checkpoint_pathway
ICOSLG	Checkpoint_pathway
ID2	T_cell_regulation
ID3	T_cell_regulation
IDO1	Drug_target
IDO2	Checkpoint_pathway
IFI27	Type_I_interferon_signaling
IFI35	Interferon_signaling
IFI44L	Interferon_signaling
IFI6	Interferon_signaling
IFIH1	Innate_immune_response
IFIT1	Type_I_interferon_signaling
IFIT2	Cytokine_signaling
IFIT3	Type_I_interferon_signaling
IFITM1	Type_I_interferon_signaling
IFITM2	Type_I_interferon_signaling
IFNA17	T_cell_receptor_signaling
IFNB1	Type_II_interferon_signaling

IFNG	Type_II_interferon_signaling
IGF1R	Adhesion,migration
IGSF6	Lymphocyte_infiltrate
IKZF1	Lymphocyte_development
IKZF2	Lymphocyte_development
IKZF3	TCR_coexpression
IKZF4	Lymphocyte_development
IL10	Drug_target
IL10RA	Lymphocyte_infiltrate
IL12A	Drug_target
IL12B	Drug_target
IL13	Cytokine_signaling
IL15	T_cell_regulation
IL17A	Helper_T_cells
IL17F	Dendridic_cell,macrophage
IL18	T_cell_regulation
IL1A	Cytokine_signaling
IL1B	Type_II_interferon_signaling
IL2	Drug_target
IL21	Cytokine_signaling
IL22	T_cell_regulation
IL23A	Dendridic_cell,macrophage
IL2RA	Cytokine_signaling
IL2RB	TCR_coexpression
IL2RG	Lymphocyte_infiltrate
IL3RA	Dendridic_cell
IL4	Cytokine_signaling
IL6	Cytokine_signaling
IL7	Cytokine_signaling
IL7R	TCR_coexpression
IRF1	Type_II_interferon_signaling
IRF4	Interferon_signaling
IRF9	Type_II_interferon_signaling
IRS1	Tumor_marker
ISG15	Type_I_interferon_signaling
ISG20	Type_I_interferon_signaling
ITGA1	Adhesion,migration
ITGAE	Adhesion,migration
ITGAL	Leukocyte_migration
ITGAM	Leukocyte_migration
ITGAX	Dendridic_cell
ITGB1	Adhesion,migration
ITGB2	Lymphocyte_infiltrate
ITGB7	Leukocyte_migration
ITK	TCR_coexpression
JAML	Lymphocyte_infiltrate
JCHAIN	B_cell_marker
KIAA0101	Proliferation
KIR2DL1	Drug_target
KIR2DL2	NK_cell_marker

KIR2DL3	NK_cell_marker
KLF2	T_cell_regulation,trafficking
KLRB1	NK_activation
KLRD1	Drug_target
KLRF1	NK_activation
KLRG1	NK_activation
KLRK1	NK_activation
KREMEN1	Neutrophil
KRT5	Tumor_marker
KRT7	Tumor_marker
LAG3	Drug_target
LAMP1	Lymphocyte_infiltrate
LAMP3	TCR_coexpression
LAPTM5	Lymphocyte_infiltrate
LCK	TCR_coexpression
LCN2	Innate_immune_response
LEXM	T_cell_differentiation
LILRB1	Leukocyte_inhibition
LILRB2	Lymphocyte_infiltrate
LMNA	Housekeeping
LRG1	Neutrophil
LRP1	Housekeeping
LST1	Leukocyte_inhibition
LY9	Lymphocyte_infiltrate
LYZ	Innate_immune_response
M6PR	T_cell_regulation
MAD2L1	Proliferation
MADCAM1	Adhesion,migration
MAGEA1	Tumor_antigen
MAGEA10	Tumor_antigen
MAGEA12	Tumor_antigen
MAGEA3	Tumor_antigen
MAGEA4	Tumor_antigen
MAGEC2	Tumor_antigen
MAPK1	Tumor_marker
MAPK14	Innate_immune_response
MELK	Proliferation
MIF	Innate_immune_response
MKI67	Proliferation
MLANA	Tumor_antigen
MMP2	Tumor_marker
MMP9	Tumor_marker
MPO	Myeloid_marker
MRC1	Dendridic_cell,macrophage
MS4A1	Drug_target
MTOR	PD-1_signaling,tumor_marker
MX1	Interferon_signaling
MYC	Tumor_marker
NCAM1	Adhesion,migration
NCF1	Chemokine_signaling

NCR1	NK_cell_marker
NCR3	NK_cell_marker
NECTIN2	Adhesion,migration
NFATC1	PD-1_signaling
NFKBIA	T_cell_receptor_signaling
NKG7	Lymphocyte_infiltrate
NOS2	Innate_immune_response
NOTCH3	Tumor_marker
NRP1	Dendridic_cell
NT5E	Checkpoint_pathway
NTN3	B_cell_marker
OAS1	Type_II_interferon_signaling
OAS2	Interferon_signaling
OAS3	Interferon_signaling
PDCD1	Drug_target
PDCD1LG2	Checkpoint_pathway
PECAM1	Adhesion,migration
PGF	Tumor_marker
PIK3CA	PD-1_signaling,tumor_marker
PIK3CD	PD-1_signaling,tumor_marker
PMEL	Drug_target
POLR2A	Housekeeping
POU2AF1	B_cell_marker
PRDM1	PD-1_signaling
PRF1	NK_activation
PSMB9	Type_II_interferon_signaling
PTEN	PD-1_signaling,tumor_marker
PTGS2	Tumor_marker
PTK7	Tumor_marker
PTPN11	PD-1_signaling,tumor_marker
PTPN6	T_cell_receptor_signaling
PTPN7	Lymphocyte_infiltrate
PTPRC	Lymphocyte_infiltrate
PTPRCAP	TCR_coexpression
PVR	Checkpoint_pathway
PYGL	Neutrophil
RB1	Tumor_marker
RORC	Helper_T_cells
RPS6	Tumor_marker
S100A8	Myeloid_marker,MDSC
S100A9	Myeloid_marker,MDSC
SAMHD1	Lymphocyte_infiltrate
SDHA	Housekeeping
SELL	Leukocyte_migration
SH2D1A	Lymphocyte_activation
SH2D1B	Lymphocyte_activation
SIT1	Lymphocyte_infiltrate
SKAP2	B_cell_marker
SLAMF7	Drug_target
SLAMF8	Lymphocyte_infiltrate

SNAI1	Tumor_marker,stemness
SNAI2	Tumor_marker,stemness
SRGN	Lymphocyte_infiltrate
SSX2	Tumor_antigen
STAT1	Type_II_interferon_signaling
STAT3	Drug_target
STAT4	Helper_T_cells
STAT5A	Cytokine_signaling
STAT6	Helper_T_cells
TAGAP	Lymphocyte_infiltrate
TAP1	Type_II_interferon_signaling
TARP	Lymphocyte_infiltrate
TBP	Housekeeping
TBX21	Type_II_interferon_signaling
TCF7	Tumor_marker
TDO2	Checkpoint_pathway
TFRC	Housekeeping
TGFB1	Checkpoint_pathway
TIGIT	TCR_coexpression
TLR3	Dendridic_cell
TLR7	Innate_immune_response
TLR8	Lymphocyte_infiltrate
TLR9	Drug_target
TNF	Checkpoint_pathway
TNFAIP8	Lymphocyte_infiltrate
TNFRSF14	Checkpoint_pathway
TNFRSF17	B_cell_marker
TNFRSF18	Drug_target
TNFRSF4	Drug_target
TNFRSF9	Drug_target
TNFSF10	Apoptosis
TNFSF13B	B_cell_marker
TNFSF14	Checkpoint_pathway
TNFSF18	Checkpoint_pathway
TNFSF4	Checkpoint_pathway
TNFSF9	Cytokine_signaling
TOP2A	Proliferation
TP63	Tumor_marker
TRIM29	Tumor_marker
TUBB	Housekeeping
TWIST1	Tumor_marker,stemness
TYROBP	Lymphocyte_infiltrate
VCAM1	Leukocyte_migration
VEGFA	Chemokine_signaling
VTCN1	Checkpoint_pathway
XAGE1B	Tumor_antigen
ZAP70	T_cell_receptor_signaling
ZBTB46	Dendridic_cell
ZEB1	Tumor_marker,stemness
