# Supplementary figures and images for: Immune Profiling of Medullary Thyroid Cancer—An Opportunity for Immunotherapy
Source: Genes (Basel). 2021 Sep 28;12(10):1534. doi: 10.3390/genes12101534 (PMC8536131; doi:10.3390/genes12101534)

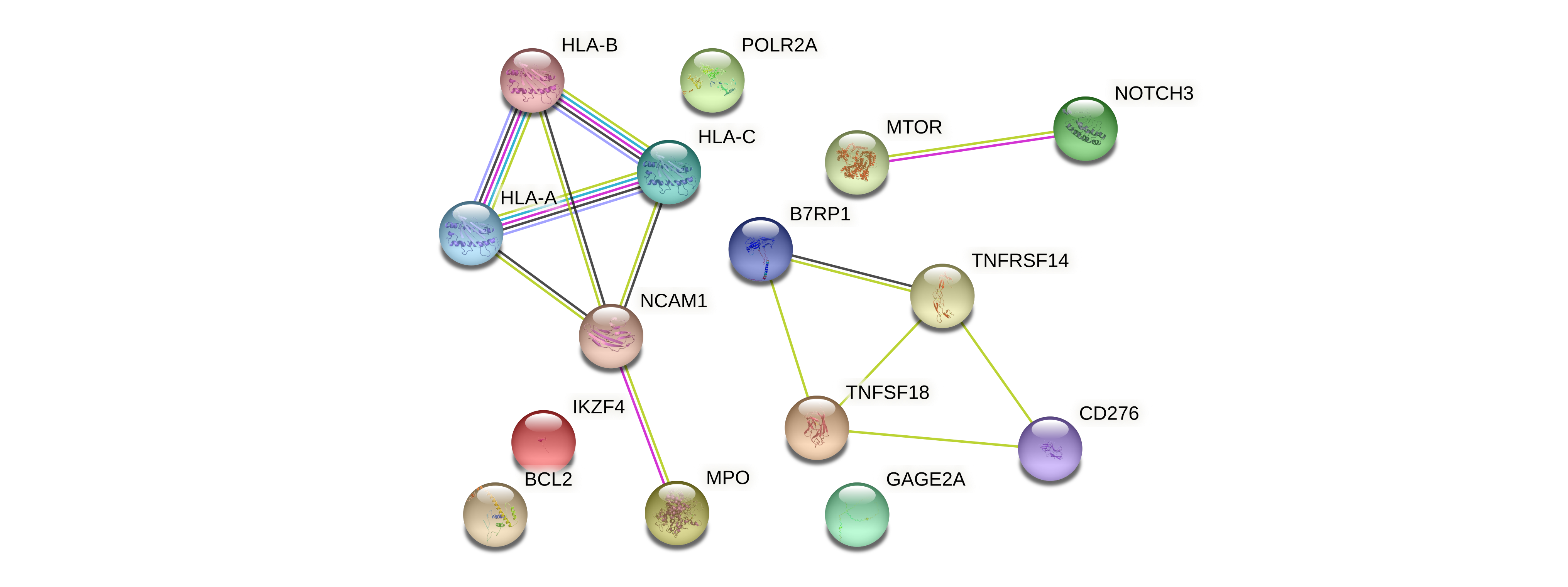

Supplement: Supplementary file 1 [file genes-12-01534-s001.zip › Figure S1_ Interaction between CD276 and other gene with more than doubled expression (string-db.org)..png]

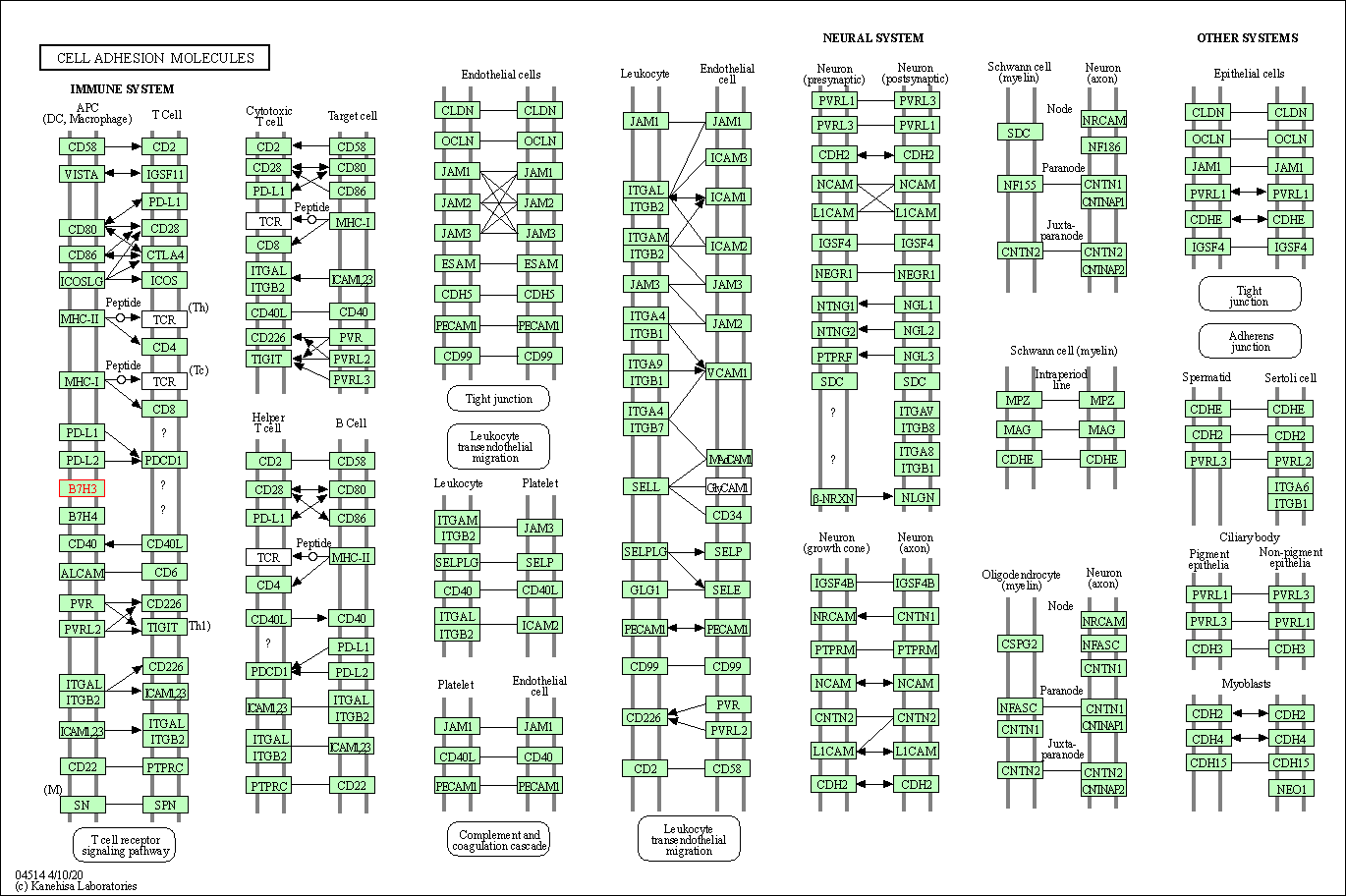

Supplement: Supplementary file 1 [file genes-12-01534-s001.zip › Figure S2_The role of CD276 (B7-H3) in T cell receptor signaling pathway (kegg.jp)..png]
